# The split ladder of policy problems, participation, and politicization: constitutional water change in Ecuador and Chile

**DOI:** 10.1007/s10784-024-09644-y

**Published:** 2024-06-19

**Authors:** Margot Hurlbert, Joyeeta Gupta

**Affiliations:** 1 Centre for the Study of Science and Innovation Policy, Johnson-Shoyama Graduate School of Public Policy, University of Regina, 2155 College Avenue, Regina, SK S4P 4V5 Canada; 2 Governance and Inclusive Development, Amsterdam Institute for Social Science Research, University of Amsterdam, Nieuwe Achtergracht, Amsterdam, 1018 WV The Netherlands

**Keywords:** Participation, Problem framing, Problem framing, Policy change, Windows of opportunity, Water governance, Water justice, Constitutional change

## Abstract

There is debate about whether complex problems should be addressed technocratically or whether they should be politicized. While many tend to favour technocratic decision-making and evidence based policy, for others politicization of policy problems is fundamental for significant policy change. But politicization does not always lead to problem solving. Nor is it always necessary. This paper addresses the question: Under what circumstances should problems be politicized, and what is the effect of such politicization? It adds politicization, through windows of opportunity, to the split ladder of participation to assess policy change through two case studies: successful and unsuccessful constitutional change in Ecuador (2008) and Chile respectively (2022). It argues that where there is no agreement on either science or policy, politicization is required to address lack of consensus in values, but constitutional protection is needed to protect minorities and the vulnerable, their access and human right to water. De-politicization stymies policy change potentially harming democracy. This paper argues for a citizen engaged exploration of the complex problem of climate change and its impacts on water, but a targeted politicization coincident with, but developed well in advance of, windows of opportunity. Moreover, policy framing correlated with complex problems continues to be a key consideration. Furthermore, alliances of disparate actors, elections of new political leaders and considerations of property rights and justice issues are paramount. Significant constitutional policy change reflects social learning, but subsequent court actions by policy entrepreneurs is required to effectively implement this change. Framing constitutional change to protect rights to water and effect international agreements (including the Warsaw International Mechanism under the climate change regime) advances water justice and may increase success.

## Introduction

Climate change is exacerbating water scarcity, drought, and lack of access to water, resulting in breaches of human rights and unattainable Sustainable Development Goals (SDGs). Central and South American countries have been experiencing severe drought conditions consistently since 2019, if not longer (Toreti et al., 2023). These national level water stresses highlight water politics and tensions in meeting international obligations for rights to water for drinking, sanitation and hygiene. Further exposed are economic considerations surrounding water as a public or private good, and processes and outcomes affecting international environmental agreements and principles of reasonable water use and participation in decision making (Mirumachi & Hurlbert, 2022). In Ecuador and Chile, water tensions have emerged in national politics and specifically constitutional amendment discussions.

Water tensions expose the “very contested, power-laden nature of water governance” (Mirumachi & Hurlbert, 2022: 1) calling for anticipating, adapting and planning for its allocation between uses and users, based on stakeholder engagement (Behnassi et al., 2019; Bosch et al., 2019). Stakeholder participation in complex socio-ecological systems (SES) reduces transaction costs, increases acceptance of adaptive strategies, trust in information and management processes and willingness to accept uncertainty and unintended consequences (Conallin et al., 2017; Jurgilevich et al., 2021; Smyth et al., 2021).

However, some scientists favour technocratic science informed policy and decision making thereby instrumentally neutralizing ideologies and power asymmetries (Saltelli & Giampietro, 2017); they mis-understand the policy process. Such technocratic solutions proposed to a captive audience of policymakers misses the mark (Cairney & Oliver, 2017). These solutions include attempts to blend natural and social science (such as adaptive management) (Conallin et al., 2017) and adaptive co-management, where they become a façade for traditional hierarchical and market based management regimes (Pearson & Dare, 2019). Problematically, complex earth and SES problems are not always regarded as holistic, interconnected SES problems, and even policymakers often advance technocratic response to policy problems (Hurlbert, 2018).

**Fig. 1:**
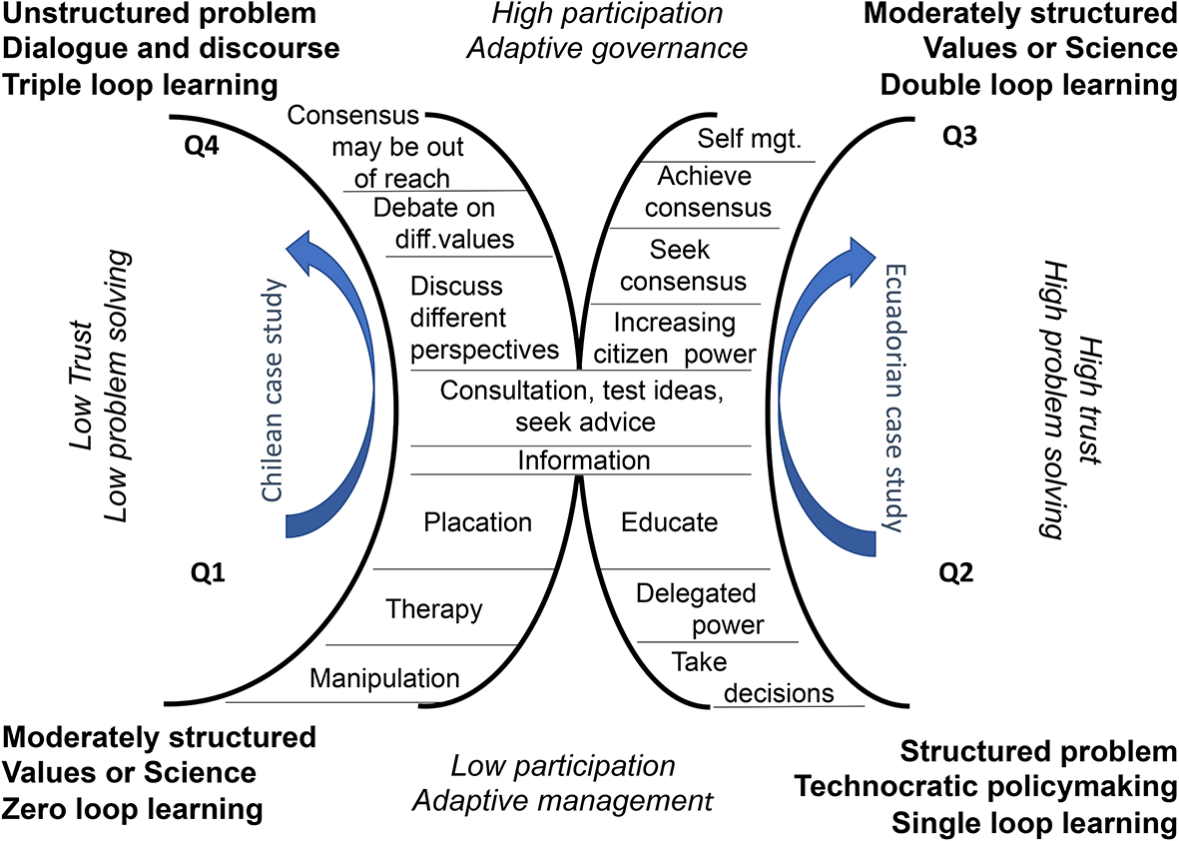
Split Ladder of Participation; source: Hurlbert & Gupta, 2015

The Split Ladder of Participation (Hurlbert & Gupta, 2015) aims to address this issue of policy framing (see Fig. 1). It conceptualizes the relevance and impacts of participation for different policy problem types (ranging from structured problems with little disagreement on science and values to unstructured policy problems with disagreement on science and values) together with differing communication styles, levels of trust, and nature of learning and governance needed. This Ladder has advanced participation science in relation to considerations of the nature and frame of policy problems (Basco-Carrera et al., 2017; Panten et al., 2018) and has been applied to transboundary fisheries (Morf et al., 2019), and knowledge production in climate, and development planning (Harvey et al., 2019; Wood et al.,. 2018).

The Split Ladder of Participation recognized uncertainty, values, and diverse constructions of issues in both problem structuring and stakeholder engagement (ranging from manipulation and placation to the possibility that consensus is out of reach). To illustrate the Split Ladder as a strategic, evaluation and diagnostic tool we used case studies which possibly advanced the ‘rationality project’ of policy sciences (Stone, 1988), recognizing the ‘bounded rationality’ of the space within which policy decision-makers operate (Fischer, 2003).

Here, we go beyond to explore the tension between addressing complex policy problems technocratically or through the subject of politicization. We ask: under what circumstances should problems be politicized, and what is the effect of such politicization? We employ the Split Ladder as a diagnostic and evaluation tool to analyze Chile and Ecuador’s water constitutional case studies to offer insights on policy agenda setting.

Politicization is the act of making an issue that was previously unpolitical, political; or transporting an issue into the field of politics (Schmidt, 2004). The Split Ladder of Participation recognizes technocratic decision making in quadrant 2; politicization conversely occurs within the center of the framework where debate, discussion, and potentially political decisions are made (Hurlbert and Gupta 2018). Not only do we envision a systems-theoretical view of politics but also our ‘framing’ of policy allows for a discourse theoretical approach (Zurn et al., 2012). While the Split Ladder has been applied in situations where issues are depoliticized, or ‘rendered technical’ (Li, 2007; for Split Ladder application Kinnunen (2021), this paper focuses on the politicization of water in the context of constitutional reform.

First, we revisit the Split Ladder and literature that has applied it, add the ideas of windows of opportunity and expand on the element of politicization in the center of the Split Ladder figure, then with a focus on water governance we review Chile’s recent attempt at constitutional amendment (2022), and Ecuador’s successful 2008 constitutional amendment. This comparative case study contributes to a theory of politicization and complex social problems.

## The split ladder and politicization

We elaborate further on the Split Ladder (Hurlbert & Gupta, 2015) in respect of politicization: (a) we expand on windows of opportunity, when problems, policies and politics come together at critical junctures resulting in policy change (Kingdon, 2011); (b) we consider how policy problems come to the attention of people in and around government, which requires a discussion of policy and stakeholders.

### Windows of opportunity

Policy scholars document incremental and path dependent policy change based on gradual evolution of policy change. Policymakers may draw lessons from existing policies in other jurisdictions or sectors for their specific context (Rose, 1993). This is typical of quadrant two of the Split Ladder when structured problems (where there is a consensus on science and values) result in technocratic policymaking; here problems are not politicized. Examples include: Dunlop and Rushton’s (2022) application of quadrant two decision making for redesigning the school curriculum and advancing climate change education in a participatory, interdisciplinary creative manner to advance learning; Potting’s (2022) analysis of science and engineering knowledge production and decision making in the seaweed industry infrastructure.

However, where there is no consensus on science and/or values, politicization is needed. This may happen through focusing events such as programme review, relicensing of infrastructure (Brian & Hannah, 2017), crises and disasters (Birkmann et al., 2008), monitoring, and/or changes in indicators (Kingdon, 2011). In these situations, rapid change and ecological crises may promote the emergence of new networks and governance forms providing a window of opportunity (Folke et al., 2005). When applying the split ladder to groundwater, Cuadrado-Quesada and Gupta (2019) conclude that meaningful participation and policy change is unlikely unless there is a ‘water crisis.’ This crisis enables discussion in the unstructured complex problem quadrant four and the possibility of policy change and social learning.

Policy changes result from a fortuitous constellation of problems, policy proposals, and politics creating a window of opportunity. First developed in 1984, this has been widely applied (Cairney & Jones, 2016). Windows of opportunity open, facilitated by policy entrepreneurs (Catney & Henneberry, 2016), or changing political landscape (Dubois & Saunders, 2017). Policy entrepreneurs push their agendas softening the system, thereby building knowledge, technical feasibility, capacity of policy makers, and public acceptance. The turnover of elected officials and an administration change can open space for changing policy. These dynamics, combined with perceived national mood, set the stage for an ‘idea whose time has come’ (Kingdon, 2011). This enables a paradigm shift (or triple loop social learning) whereby policy changes as a result of change in values and assumptions (Prutzer et al., 2021).

### Policy and stakeholders

While the Split Ladder conflated actors and policies (however, expanded in Hurlbert et al., 2019), this article widens the discussion. While the policy ecology is metaphorically described as a garbage can model of organizational choice (Cohen et al., 1972), others describe it as a ‘policy primeval soup’ in which specialists try out combinations of ideas from a smorgasbord of policies (Kingdon, 2011). Policy proposals survive when they meet criteria from technical feasibility, fit with dominant values and current national mood, budgetary workability, and political support (ibid.).

Policies depend on the support and attention of policy actors. Elected officials, and bureaucrats under their direction, make policy by agenda setting, specifying alternative policy choices, legislating and implementing decisions. Policy change occurs through interactions between theorists who propose ideas, framers who transmit ideas like gatekeepers (bureaucrats, academics), constituents representing public sentiments (voters) and brokers who transport ideas (acting as public relations experts, advisors, think tanks and epistemic communities)(Campbell, 2004). This can happen through advocacy coalitions of actors from different institutions who share basic beliefs and jointly propose policies (Sabatier & Jenkins-Smith, 1993) possibly based on narrative storylines (Hajer, 2005). Since political contestations deliver policy change, policy may not be congruent to surveyed public opinion (Kinnunen, 2021).

Water and climate governance is inherently political as it brings together conflicting values (such as support or opposition to fossil fuel development (Roth et al., 2017) or competing human rights to water (Robina Ramírez & Sañudo-Fontaneda, 2018). Depoliticizing such complex issues reduces trust in government and harms democracy (Roth et al., 2017; Dunlop et al., 2021; Hoogesteger, 2012). To assess quadrant four of the Split Ladder of Participation and policy change, this paper compares Ecuador and Chile’s attempts at constitutional change.

## Case study method

We use a qualitative comparative case study to understand when problems should be politicized, and the effect of such politicization. We study two cases of (un)successful constitutional change to explore the politicization and the mechanisms of change. Here constitutional change is synonymous with policy change defined as, “a course of action or principle adopted or proposed by a government, part, business, or individual” (OECD 2008: n.p.) and a recognized heuristic, complex, unit of case study analysis of legal change (Husa, 2020).

We present a narrative of decision processes of national constitutional change (Yin, 2017; Jensen and Rogers 2001). While the dependent variable is constitutional water politicization, the independent variables are institutional and structural dimensions of citizen engagement, emergent framing narratives (Schmidt, 2010), and consequences (or outcomes); these dimensions influence the flow of ideas, influence and actions (Parreira do Amaral, 2022; Bartlett & Vavrus, 2017). The narratives are based on a literature review, secondary sources, constitutional texts and proposals, and relevant submissions or positions of interested parties.

### Two constitutional water cases

#### The chilean water constitution case

South America in general, and Chile in particular, is suffering from the impacts of climate variability and change especially through glacier mass loss worldwide (20–60% in the Andes since 1985), extreme floods and droughts, and resulting impacts on agriculture (Castellanos et al., 2022). The ‘Central Chile Mega Drought’ entered its thirteenth year in 2022; central Chile has had a 76% deficit in rainfall in the 2020s of a ‘normal’ year. This is the longest drought in a thousand years (Zyri 2022; UN, 2022). Competition over water may worsen and degrade ecosystems putting 31 million people in water stress in South America (Castellanos et al., 2022). Falling rain levels by 20–40% since 2010 and atrophying glaciers and aquifers have made Chile a top country facing water stress (World Resources Institute, 2015).

The 1981 Pinochet coup ushered in a Constitution that privatized water, granting perpetual property rights allowing powerful mining, energy and export agriculture interests to usurp community drinking water and traditional local water practices (Hurlbert, 2018). It promoted an export economy of large scale irrigated agri-business producing high-value crops like fruits, vegetables and grapes (Reyes, 2009) advancing a narrative of user pay and full water cost at the expense of human rights to water (Larrain, 2014). Agriculture now uses 72% of water followed by drinking water (12%), industrial consumption (7%), hydroelectric/grazing (5%) and mining (4%) (Ramirez, 2022).^1^ Export agriculture has meant wealth for some in Chile. In 2021, Chile’s real GDP was $25,400 per capita and the Gini index score of 44.9 situates Chile as a leading South American country (CIA 2023).

And yet, 8% of the population don’t have access to water to fulfil their human rights Langrand et al. (2022) and rural communities compete with large companies for water and are hit the worst. While a single avocado exported to North American and European supermarkets takes 320 L of water, some people only receive 15–20 L per day (flushing the toilet takes 10 L) (Langrand et al., 2022).

Chilean water governance is dominated by its privatized market water interests (Hurlbert & Diaz, 2013) but attempts to protect vulnerable people and the ecosystem have occurred (including protecting ecological flows and a levy for unused water rights to prevent hoarding)(Hurlbert, 2018). However, such attempts have little impact on prior constitutionally protected water owners (Hurlbert, 2018). In times of drought (defined as a river flowing at less than 70% of average) the Chilean Water Code (Article 314) allows irrigators to request a Presidential Declaration of Drought Zones. Upon Declaration, proportional reduction of water to each rights holder in turns is employed for a maximum of six months (Hurlbert, 2018).

After the Chilean plebiscite to return to democracy in 1988, the government severed links with social movements for fear of social unrest returning Chile to an autocratic state (Somma, 2017). However, early this Century, discontent with markets and political elites resulted in student protests (against expensive higher education), environmental protests (against externalities of forestry, energy and mining companies), and Indigenous protests (such as the Mapuche against the impacts of development on their lifestyles and environment) (Somma, 2017). Recently, the number of protests and participants has increased steeply (Larsson, 2019).

#### The window of opportunity

In 2019 a million protesters demanded President Pinera’s resignation (Larsson, 2019) and a new constitution (McGowan, 2019). Protests were linked to the rise in metro fees.^2^ Other reasons included lack of education, poor public health care, and crippling inequality experienced through increasing costs of living, low wages and pensions. Chile embarked on drafting a new constitution after President Gabriel Boric defeated conservative Jose Antonio Kast in the 2021 election.

Since political exclusion fuelled the protests, Chile’s constitutional convention (2021–2022) included nationwide deliberative meetings, local town hall meetings, self-convened meetings by citizens, consultation with Indigenous peoples, and a digital public participation platform. Further, public hearings received hundreds of expert and citizen submissions. Public constituent hearings also occurred with people in the different Chilean constituents. 2486 citizen initiatives were supported by 220,000 people with ten gaining enough support to be discussed by the Convention including issues of education, religious freedom, animal rights, and nationalization of mining companies (Fuentes, 2022).

In September 2022, Chile’s constitutional assembly finalized a new Constitution of 388 articles. It replaced the Senate with a ‘Chamber of Regions’, decentralized taxing powers to regions, provided social services (healthcare, housing, education), and switched water and resource rights to temporary revocable permits (Burns, 2022). It defined Chile as a ‘pluri-national’ state including the customary law of Indigenous people and declaring water rights ‘*incomerciables*’ or ‘unsalable.’ These provisions were however unclear (ibid.) and the constitution was rejected (Surma, 2022). Reasons cited include fake news orchestrated by well-organized negative campaigns (Fuentes, 2022), mandatory voting of all citizens (while many had not participated) (People Powered, 2022), early term poor performance of Boric’s government and its failure to tackle rising crime and terrorism concerns, too much change from the 1981 Constitution, inflation, and a sluggish economy (Rodriguez, 2022). Instead of uniting people, the rejection of the new constitution possibly reflected a rejection of the process, as the elected representatives of the Constituent Convention were delegitimized having lost citizen trust in their ability to guarantee an adequate framework. The constitutional failure was also seen as a rejection of President Boric’s performance (Edelman Gobal Advisory 2022). Since then, a new constitutional writing plan commenced. Drafters included 50 members of an elected Constitutional Council; 24 experts and 14 jurists formed the ‘Admissibility Technical Commission’ and acted as arbitrator (Nodal, 2022). This newest constitutional endeavor also failed with 56% of the electorate voting against it in December 2023 (Villegas, 2023).

Meanwhile, a new water code has been passed which prioritizes water for human consumption and allows government to temporarily suspend usage rights on threatened watercourses (Langrand, 2022; Ramirez, 2022). Water permits will be granted under a 30-year renewable concession (or less) based on water availability or Aquifer sustainability; unused water permits can be revoked (Ramirez, 2022). Registration of water rights is mandatory (only 4% are presently registered). This code excludes the 90% of water rights already granted (Langrand, 2022). As a result, uncertainty surrounding water shares continues within Chile (Ramirez, 2022).

#### Ecuador

*Glacier* loss, precipitation variability and land use changes have impacted livelihoods and water resources (Castellanos et al., 2022). ENSO causes heavy rains, storms, floods, landslides and increasing heat waves and consecutive dry days; this trend may increase in the future (ibid.). With natural assets including the Galapagos and Amazon, Ecuador has led the world on equity and sustainability in its laws and policies, (but suffers economic and functional challenges in implementation) (Wingfield et al., 2021). Ecuador’s per-capita GDP is $10,700 (less than half of Chile’s) and its Gini Index is 47.3 (more unequal than Chile) (CIA 2023); per capita water availability is three times the international average (Instituto Geografico Militar 2013). While 60.3% of rural people use safely managed sanitation, only 31.2% of urban people do (UN, 2023). While 74.7% of urban people use safely managed drinking water, only 52.8% of rural people do (ibid.). Poor technical and infrastructure capacity challenges water management; moreover, decentralized drinking water and irrigation management conflicts with the national responsibility for water control and management (Wingfield et al., 2021). Ecuador has a decentralized administration of 24 provinces (van den Berg & Danilenko, 2019).

Pre-1960, water privatization was allowed with water viewed as a commodity rather than a public good (Martínez-Moscoso et al., 2018). Between 1970 and 1990 Ecuador’s water laws advanced decentralized and privatized state resources (Cremers et al., 2005), but water was declared a public good in the early 1990s effectively ending privatization (Martínez-Moscoso et al. 2019). But privatization again emerged with new public management in the 1998 Constitution. The state’s role was minimized and private irrigation management prioritized (Law of Decentralization and Irrigation Management Transfer programmes; Hoogesteger et al. 2013). This process was aided by the development of oil exports and the predominant influence of these companies (Shade, 2015; Mena-Vásconez 2017) and Chinese investment (Ray and Chimienti 2017). An export economy reliant on water-intensive floriculture production (roses) has developed (Damonte, 2019), paying one quarter of the water rate of community users (Hidalgo et al., 2017). Further, the principle of *buen vivir* has successfully been argued in court to protect the mining livelihood of small scale gold miners. These successes have resulted in environmental protestors of mining expansion being prosecuted criminally. As a result, the argument of *buen vivir* has protected a mining livelihood at the expense of the environment (Valladares & Boelens, 2019).

The window of opportunity.

In the early 2000s a window of opportunity opened when people protested privatization and neoliberal reform. Since the 1980s, Indigenous and environmental organizations opposed the exploitation of oil, water, and precious metals protesting pollution, deforestation and the use of genetically modified organisms. Rural people fought against privatization of oil, electricity and telecommunications in the 2000s and momentum grew (Dosh and Kilgerman 2009). In 2007, thousands of people lobbied for a new constitution to be drafted by the Constituent Assembly and its 1,230 delegates. The draft document had 444 articles.

This draft constitution moved away from privatization (Asamblea Nacional, 2014). Water was declared a human right in 2008 (Asamblea Nacional Constituyente 2008). It recognized the rights of ‘Pachamama’ or Mother Earth. It incorporated *sumac kawsay* harmonizing human development and environment protection by recognizing the innate rights of nature and *buen vivir* to improve living conditions (Kauffman et al. 2014, Tanasescu, 2013). The window of opportunity partly arose due to the resistance to neoliberal privatization policies (Hoogesteger, 2012). Over 60% of voters backed President Correa’s new constitution; Correa was elected to fight long standing corruption, ban foreign military bases, allow same sex marriage, and greater national control over oil and mining industries (Britannica, 2023). This was a “moment of unity between Ecuador’s popular movements and the electoral left” (Dosh & Kligerman, 2009: n.p.).

Popular movements clarified that their support for the Constitution in 2008 wasn’t support for the President (Dosh & Kligerman, 2009). Subsequent clashes over mining through 2008–2009 demonstrated that these groups had different interests (ibid.) and effecting the constitutional change in relation to water did not happen immediately. In fact, the Constitution allowed an exception to enable privatizing natural resources and water, allowing the president to give permission to extract resources. This was dubbed by the former government communications secretary as “one of the biggest deceptions of the Constitution” (ibid.). The deceptive rules allowing Presidential exceptions were incongruent with the *minga* model granting access to community members due to their work rather than a payment (Hoogesteger, 2012), a practice that informs local community water management to this day (Hoogesteger, 2013, Wingfield 2021).

Widespread demonstrations after 2008 protested a new mining law to allow Canadian mining corporations to begin operations (Dosh and Kilgerman 2009). On the day of the Mining Law’s passage, 4,000 Indigenous people blockaded a highway in the south and tens of thousands mobilized in Quito, Cuenca, the Amazon and on the coast (Zibechi, 2009). The President of Acción Ecológica in 2009 argued that the state would not be able to protect natural resources and that the President had ‘politicized’ the problem by stating that his principal enemy was ecologists (Dosh and Kilgerman 2009). Other protests included the *Caminata del Agua* movement of 2014 and those led by Carlos Perez Guartambel (*Yaku*) protesting mining activities near water resources (Kolirin, 2020). These protests centered on water, but also larger protests occurred on entry requirements impeding hundreds of thousands of students attending university, labour unions and teachers against anti-union prohibitions, indefinite re-election laws for the President, and against the continuous criminalization of social protest (Picq 2014). These local demonstrations garnered some success in the courts including prevention of expansion of an access road that would have increased contamination risks to the Vilcabamba River in 2011 (Dupuits et al., 2020).

In 2014 water was declared a national strategic asset for public use and privatization effectively outlawed (Asemblea Nacional 2014). Local community or individual water management is allowed pursuant to this law in the event of finance and construction of the main water infrastructure (Asemblea Nacional 2014). Municipal governments must provide potable water, and sewage and wastewater treatment and are forbidden from suspending these services (Art 264(4); 326(15) Asemblea Nacional 2008). However, this is subject to the Water Secretary’s authority and national regulative body that prioritizes use as human consumption first, irrigation that guarantees food sovereignty, ecological flow, and lastly productive activities. This has reduced the role of private companies in water management.

Ecuadorian courts have embraced the constitutional water laws prioritizing them over mining and development. They have implemented the rights of nature laws in a ruling in favour of mangroves and also Los Cedros protected area’s right to flourish (thereby stymying mining activities). The court recognized the inherent value of nature that must be recognized in and of itself without consideration of ‘usefulness’ for people (Surma, 2021). Further, a September 2020 ruling of the constitutional court halted mining activity in the Cuenca region (Primicias, 2020). While constitutional change occurred in 2008, its consolidation in Ecuadorian law and practice was realized in the ensuing years.

### Analysis

#### Policy problem framing

Where there is agreement on science and values, there is no need to politicize an issue as single loop learning is adequate. Prior to Ecuador’s constitutional reform, water was managed in a privatized, decentralized technocratic manner advancing irrigation interests. Chile had administered its water resource within quadrant two of the Split Ladder of Participation where water rights are private property interests protected within legal institutions in a technocratic manner. Both case study countries’ windows of opportunity arose through a combination of policy problem framing and participatory contestation or politicization. These case studies demonstrate that the window of opportunity for significant change involved complex multi-faceted political problems.

Table 1 displays the policy problem framing in Chile and Ecuador within the text of the constitutional amendment and the resulting constitutional changes (effected in Ecuador and defeated in Chile). While Chile’s predominant framing was in relation to addressing social inequality and the high cost of living, Ecuador’s centered on advancing a liberal agenda, good governance (anti-corruption), and national control of oil and mining. Overturning water privatization was not the predominant problem framing for Ecuador.

**Table 1:** Policy problem framing and constitutional provisions in chile and ecuador

	Chile		Ecuador	
	Problem Framing	Constitutional Framing	Problem Framing	Constitutional Framing
Rights	Estallido Social (Social outburst)	Rights – 109 articles (dignified life, democracy, peace and the balance of nature, endowing people, Indigenous nations and nature as holders of individual and collective rights)	Advance liberal agenda	Rights – 9 chapters (*buen vivir*, persons/groups, peoples, participation, freedom, nature, protection, responsibilities)
Participation	Reduce cost of living	Democratic participation	Anti-corruption	Participation and organization of power
Good governance		Good governance and public function		The good way of living system (inclusion and equity, biodiversity and natural resources)
State organization		Regional state and territorial organization		Territorial organization of the state
Government	Reduce inequality	Government (legislative and executive)	National control of oil and mining	International relations (principles, treaties, Latin American integration)
Justice		Justice systems		Development structure (participatory planning, food sovereignty, economic sovereignty, strategic sectors, services and state enterprises, labor and production, commerce and fair trade, savings and investment)
Constitution		Autonomous Constitutional bodies (Central Bank, General Comptroller etc.)		Supremacy of the Constitution

As we (Hurlbert & Gupta, 2015) originally concluded, exploration, discussion and participation in solving complex problems results in the creation of policy sub-problems, the combination of which may address the complex problem. The Chilean constitutional renewal was laudable, but it proved to be an insurmountable task. Where enduring patriarchical institutions, widening social inequality, looming climate change impacts of drought and fire were ravaging the Chilean landscape, remedial hopes with one participatory constitutional engagement were laudable, but unrealistic (The Economist, 2022). The Ecuadorian ‘problem’ included foreign exploitation of oil and mining effectively addressing inherent contradictions of sustainable ‘development’ and the environment (Reid, 2023); this problem framing was missing in Chile.

#### Policies and actors

In both countries the window of opportunity also arose due to the election of a new President with a new political agenda; each embarked on renewing the constitution embracing a quadrant four politicization. For both countries politicization was needed, but in Chile, as is often the case, the problem was not solved in the short term (Smyth et al., 2021). In Chile, the complex water problem arose due to both the omnipresence of climate change (and the mega drought) and the widespread malaise surrounding Chile’s constitutionalized private water market.

If only symbolic participation is advanced, and there is consensus on either science, or values, but not both, politicization will lead to non-decisions and reproduction of injustices. In Chile, the consensus on mega drought science is irrefutable, but the symbolic participation in the Constitutional amendment foreshadowed its failure (People Powered, 2022). Although the scope and ambit of the constitutional renewal process was good, COVID reduced participation. Hence, Chile’s endemic social problems are jettisoned back to a problem of mega drought adaptation science. Here zero loop learning plays out as historic processes continue to be employed in water reductions, perpetuating Chilean water injustice (lack of poor people’s access to water), and without addressing the root problem. Constitutional protection of a human right to water is missing.

The Ecuador case study reflects successful policy solutions and social learning where complex problems were reduced to broad constitutional reform principles (see Table 1). Ecuador’s 2008 Constitutional amendments advanced Nature’s rights, showing how complex problems are reduced to lesser sub problems, and addressed in a productive manner moving into quadrant three increasing citizen power. While consensus on water and development issues was not immediately achieved with the constitutional reform, citizens obtained their human rights to water and the creation of Nature’s rights to advance consensus through legal remedies. The Ecuadorian case importantly illustrates that mere Constitutional textual reform, while it appears as significant ‘change,’ may not result in actual change. Policy change should be carefully defined as ‘radical’ change where overarching terms of policy discourse associated with paradigmatic shift occur and a wholesale change in ‘ideas’ and social interests (Hall, 1993).

Engagement in complex politicized problems in quadrant four is no easy task; meaningful involvement of social scientists is paramount and in respect of restoring freshwater ecosystem services in the context of local and global change, policy change, paradigm shift and significant social learning may not be feasible in the short term (Smyth et al., 2021). The Chilean case illustrates the tension of requiring a high level of value consensus for constitutional change to protect the vulnerable. While the Ecuador case contained value consensus of protecting national interests in oil and mining and pushing back on privatization of water, the Chilean case did not. While the Ecuador case involved consensus of actors for the constitutional solution (but not necessarily their endorsement of the President), the Chilean case could not achieve this.

These two case studies do not necessarily support the conclusion that the Ecuadorian value against privatized water must exist for triple loop learning and water law reform. In Sharma and Kumar (2020) the Split Ladder analysis of water, energy and food confirmed a two-step process for consumers and producers whereby the market dictated the first step in the process of decision making for water access and allocation, but then the triple rights of people to water, energy and food determined the second step in the allocation and access decision making distributing these assets. It may be that in Chile, such a two-step process is more fitting and palatable.

Procedural justice and power in politicization are fundamental considerations. Holistic power analysis that advance understanding of contextual factors that favour stakeholder participation increase success (Wood et al., 2016). However, politicization of issues may exclude the interests of the minority, including Indigenous peoples. Addressing these concerns may not occur without successful Constitutional amendment to protect minority rights. Chile’s Water Code amendments are a start, but lack the power of constitutional status protecting recognized Chilean rights. Continued politicization of Indigenous and water rights and participation of people in decision making through all avenues, including legal ones are required. In the Ecuador case study the 2008 and 2014 constitutional reforms and subsequent legal actions illustrate this point. In Ecuador Indigenous people continue to oppose Presidential decrees to increase oil production and pipelines in their lands and advance their constitutional rights including the requirement for their consent prior to development initiatives (Waorani Pastaza Organization vs. Ecuador 2019; A’I Kofan community of Sinangoe vs. Ecuador 2022).

Policy windows are essential for double or triple loop learning. The multiple streams approach has primarily been applied to analyze a policy change after the fact. Thus, policy windows are often viewed as an opportunity rather than intentionally created. Hence, while literature has documented strategies of policy entrepreneurs to advance policy change and prepare for a policy window (Brouwer & Biermann, 2011), there is scant literature documenting methods that have intentionally and successfully created policy windows (Hermansen, 2015). While Chile’s new Constitutional Council (commencing in 2023) will have a window of opportunity to make significant change through double or triple loop learning, its success will depend on its policy entrepreneurs (actors) and their ability to set a water policy agenda. The Split Ladder of Participation provides a framework to analyze complex policy problems and prospectively set agendas for participation advancing their resolution.

## Conclusion

This article interrogates significant policy change and public participation in relation to constitutional change. Politicization doesn’t always lead to problem solving, but it can open a window of opportunity for change and be important for a healthy resilient democracy. This paper addresses the question: Under what circumstances should problems be politicized, and what is the effect of such politicization? It builds on the split ladder of participation to develop a theory on politicization of complex societal problems.

This paper explores people’s participation in solving complex policy problems through problem framing and windows of opportunity. While responding to complex problems through public engagement and politicization is often touted as optimal, this paper explores how problem solving is best achieved in relation to significant policy change – specifically constitutional reform. Two case studies provide illustration: the successful 2008 constitutional reform of Ecuador and the unsuccessful 2022 Chile attempt at constitutional reform. This paper argues for a holistic exploration of a complex problem such as water governance, which is often situated in relation to overarching policy problems of development and inequality. Policy entrepreneurs are well advised to continue to advance needed reforms creating and seizing the moment afforded by windows of opportunity; our conclusion is that politicization is required for significant policy change and agenda setting, and recommended.

When there is agreement on science and policy, quadrant two suffices and politicization is not needed. Policy systems are advanced enough to repair and modify instruments to address problems. Where there is no agreement on either science or policy, politicization may be needed to address lack of consensus in values (Islam & Susskind, 2018), but cannot be sufficient. Here constitutional protection is needed to protect minorities and the vulnerable and avoid the reproduction of injustices. Inadequate politicization (and accompanying participation of people and civil society as in the Chilean case) can lead to zero order learning and the reproduction of injustices. In turn policy windows can close with growing distrust. Participation in water decisions is most importantly informed by ecological and social inclusion through considerations of human dignity and equality, rather than legal and economic considerations (Pouw & Gupta, 2017).

Stakeholder participation and politicization do not guarantee that minority rights, or rights of nature will be protected. Such protection needs to be guaranteed by constitutions at the national and global level. Politicization and constitutional discussions are also preconditions for addressing the increasing interconnections of water scarcity, drought, and groundwater between states and normative principles of equitable and reasonable use and participation within Article 5 of the United Nations Watercourses Convention. The 2010 recognition of the human right to water and sanitation by the UN General Assembly (UN, 2010) enabled human rights claims as a legal strategy to advance climate adaptation and mitigation efforts; courts are becoming increasingly receptive (Peel & Osofsky, 2015). Such claims can be anchored in the Warsaw International Mechanism under the climate change regime or before the United Nations Human Rights Council and the Office of the High Commissioner for Human Rights. However, such claims are not assured, given the advancement of water futures trading on Wall Street: since 2020 investors can hedge against or for the potential of water scarcity effectively transforming citizens into clients (Fox, 2020).

While national constitutional protection is easier than global protection, the Ecuador case demonstrates that constant vigilance through participation, including in the legal system is still required. And perhaps, advancing constitutional change to align a nation’s law with international legal obligations would increase the success of constitutional water initiatives. Although constitutional reform can promotedoctrinal legal change, it is not until behavioural assumptions and social relations change, that a paradigmatic change has occurred (Hall, 1993; Daigneault, 2014). Policy change is not only required, but also demonstrated change in values, conceptions of what the problem actually is, ideas about which policy ends to pursue and appropriate policy means to achieve those ends (Daigneault, 2014) all of which may take time. As we are moving into the world of increasingly unstructured problems, we need overall values that both bind us and empower us to live in a safe and just world. And we need statesmanship; these cases demonstrate the importance of new political leaders.

The limitations of this research are that it is contextualized, case study research. More case studies, with different policy problems, different levels of study (international, sub-national etc.), and geographies need to consider these issues and depoliticization in the context of the Split Ladder, or policy change and citizen engagement. While deep insights are provided in this comparative case study, more studies exploring these phenomena would advance our understanding of when politicization is needed and what are the effects of politicization.

## Abbreviations

Gini Index: Measure of inequality proposed by Corrado Gini
SES: Socio-ecological systems
UN: United Nations

## Footnotes

1 A counter narrative is provided by over 1900 rural water cooperatives, many existing since 1964, often headed by women that provide drinking water for nearly two million rural inhabitants (Langrand et al., 2022).

2 Students, however, had been protesting over the past decade (Larsson, 2019) over high tuition.
